# Analysis of 2′-hydroxyflavanone (2HF) in mouse whole blood by HPLC–MS/MS for the determination of pharmacokinetic parameters

**DOI:** 10.3389/fchem.2023.1016193

**Published:** 2023-03-09

**Authors:** Luiza F. O. Gervazoni, Gabriella Gonçalves-Ozorio, Taiana Ferreira-Paes, Aline C. A. Silva, Gabriel P. E. Silveira, Heliana M. Pereira, Douglas P. Pinto, Edézio F. Cunha-Junior, Elmo E. Almeida-Amaral

**Affiliations:** ^1^ Laboratório de Bioquímica de Tripanosomatídeos, Instituto Oswaldo Cruz, Fundação Oswaldo Cruz, Rio de Janeiro, Brazil; ^2^ Laboratório de Farmacocinética, Fundação Oswaldo Cruz, Rio de Janeiro, Brazil; ^3^ Laboratório de Imunoparasitologia, Unidade Integrada de Pesquisa em Produtos Bioativos e Biociên-cias, Universidade Federal do Rio de Janeiro, Campus UFRJ, Macaé, Brazil

**Keywords:** HPLC-MS/MS, pharmacokinetics, 2′-hydroxiflavanone, flavonoid, oral treatment

## Abstract

Given the lack of investments, structure, and difficulty of metabolite isolation, promising natural product studies do not progress to preclinical studies, such as pharmacokinetics. 2′-Hydroxyflavanone (2HF) is a flavonoid that has shown promising results in different types of cancer and leishmaniasis. For accurate quantification of 2HF in BALB/c mouse blood, a validated HPLC-MS/MS method was developed. Chromatographic analysis was performed using C_18_ (5μm, 150 mm × 4.6 mm). The mobile phase consisted of water containing 0.1% formic acid, acetonitrile, and methanol (35/52/13 v/v/v) at a flow rate and total running time of 0.8 mL/min and 5.50 min, respectively, with an injection volume of 20 µL. 2HF was detected by electrospray ionization in negative mode (ESI-) using multiple reaction monitoring (MRM). The validated bioanalytical method showed satisfactory selectivity without significant interference for the 2HF and IS. In addition, the concentration range between 1 and 250 ng/mL showed good linearity (r = 0.9969). The method showed satisfactory results for the matrix effect. Precision and accuracy intervals varied between 1.89% and 6.76% and 95.27% and 100.77%, respectively, fitting the criteria. No degradation of 2HF in the biological matrix was observed since stability under freezing and thawing conditions, short duration, postprocessing, and long duration showed deviations less than 15%. Once validated, the method was successfully applied in a 2HF oral pharmacokinetic study with mouse blood, and the pharmacokinetic parameters were determined. 2HF demonstrated a C_max_ of 185.86 ng/mL, a T_max_ of 5 min, and a half-life (T_1/2_) of 97.52 min.

## 1 Introduction

Flavonoids are present in fruits, vegetables, spices, teas, coffee, and wine; are a large complex of secondary metabolites; and are used on a large scale by humans worldwide. The large complex is divided into subclasses classified as flavans, flavanones, flavones, isoflavones, isoflavans, isoflavanones, flavanols, isoflavanols, neoflavonoids, dihydroflavonol, flavan-3-ol, flavan-4-ol, rotenoids and pterocarpans (Hoensch and Oertel, 2012; Gervazoni et al., 2020; Ullah et al., 2020). Among the different effects of flavonoids on living organisms, strong antioxidant potential and anti-inflammatory power stand out for controlling diabetes, cardiovascular diseases, cancers, and several other diseases (Hsiao et al., 2007; Nagaprashantha et al., 2011).

**FIGURE 1:**
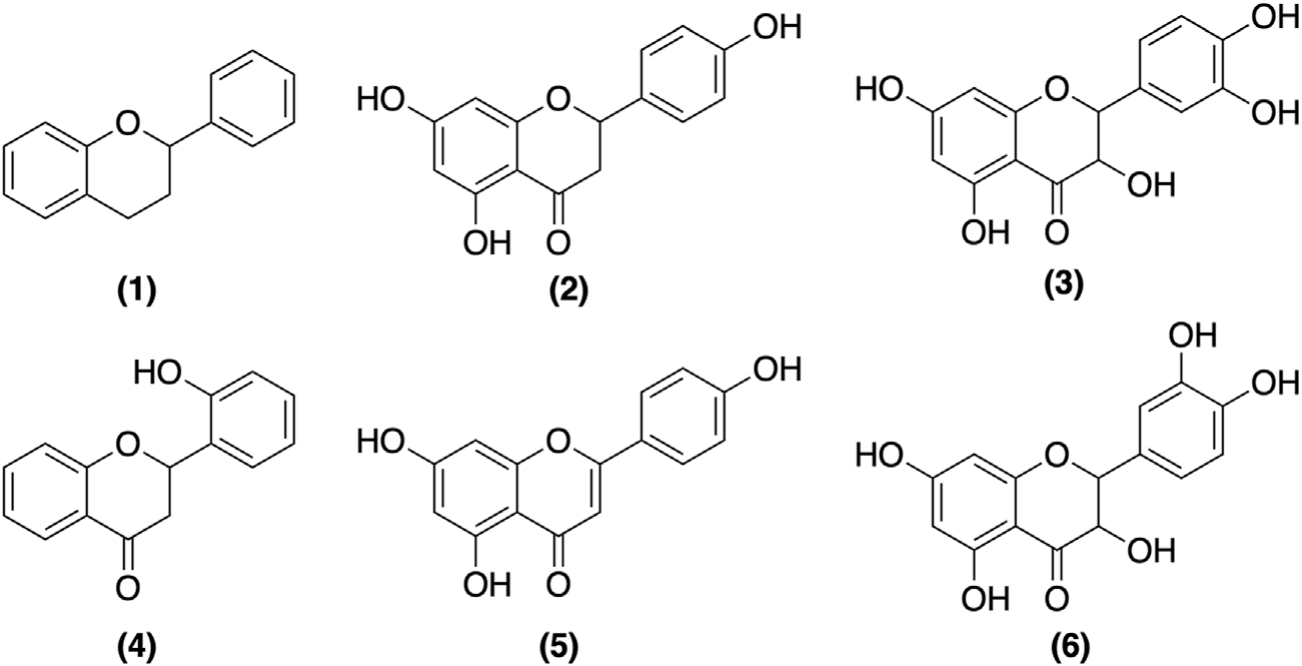
Chemical structures of representative Flavonoids (1) Flavonoids basal structure (2) Naringenin—flavanone (3) Taxifolin—flavanonol (4) 2′-hydroxyflavanone—flavanone (5) Apigenin—flavone and (6) Quercetin—flavonol.

2′-Hydroxyflavanone (2HF) is a flavanone and is mostly found in the peels and seeds of citrus and the leaves, stems, and roots of various plants. A large study on 2HF has been conducted in the oncology field, showing that 2HF is promising in the treatment of different types of cancer with different mechanisms of action. These mechanisms can prevent the proliferation and vascularization of tumors and even the differentiation of tumor mutants (Hsiao et al., 2007; Nagaprashantha et al., 2011; 2018; 2019; Shin et al., 2012; Singhal et al., 2015; Bose et al., 2019; Yue et al., 2020). The structure of 2HF and flavonoids of different subclasses are presented in Figure 1.

Recently, our group demonstrated the ability of 2HF to inhibit both the promastigote and amastigote forms of *Leishmania amazonensis in vitro*, decrease the lesion size and almost cease the parasitic load in a sensitive and cutaneous murine model of leishmaniasis, suggesting that 2HF is a potential candidate for leishmaniasis treatments (Gervazoni et al., 2018).

Even though natural product drugs have been used since ancient times and have many known properties, developing drugs from natural products is still difficult and is occurring less frequently, as semi and synthetic derivatives are now being developed (Patridge et al., 2016). For trypanosomatid diseases, for example, several plant metabolites, including flavonoids, have been described as promising drugs; however, over 90% of them do not make it to drug development phases, which may be due to the lack of incentive, their technological structures, or the difficulty of isolating the active metabolite (Schmidt et al., 2012a; Schmidt et al., 2012b; Thomford et al., 2018; Beutler, 2019).

The drug development process comprises different phases, including pharmacokinetic studies, which is an essential parameter for explaining the bioavailability and distribution of drug candidates that possess high pharmacological potentials. The development of a sensitive, specific, and validated method for detecting compounds in total blood or plasma is an important and limiting step in the process, especially for natural products such as crude extracts or isolated metabolites (Ou-yang et al., 2013; Huang et al., 2017). Several studies have highlighted HPLC–MS/MS (high-performance liquid chromatography coupled with tandem mass spectrometry) for flavonoid pharmacokinetic determination because of its ability to perform a qualitative and quantitative analysis with high sensitivity and selectivity in different biological matrices, indicating also the importance of choosing the best extraction method since it discards any interference of matrix components (Ou-yang et al., 2013; Huang et al., 2017; Attwa et al., 2018; Chavari et al., 2019; AlRabiah et al., 2020; Yuyama et al., 2020). Hesperidin, naringin, naringenin, quercetin, and luteolin are examples of flavonoids with pharmacokinetic properties that have been described by HPLC–MS/MS (Xu et al., 2018; Araujo-León et al., 2020).

This work aimed to develop and validate an efficient bioanalytical method based on HPLC–MS/MS for accurate quantification of 2HF in BALB/c mouse blood and apply it in a pharmacokinetic study, determining the pharmacokinetic parameters.

## 2 Materials and methods

### 2.1 Compounds and reagents

2′-Hydroxyflavanone (≥98% purity; lot SLBT8413), naringenin (≥95% purity; lot BCBB6396), and dimethyl sulfoxide (DMSO) were obtained from Sigma–Aldrich (St. Louis, MO, United States). Methanol was obtained from Merck (Darmstadt, Germany). HPLC acetonitrile was purchased from J.T. Baker (New Jersey, United States). Formic acid 96% and tert-butyl methyl ether (TBME) were purchased from Scharlab (Barcelona, Spain). Ora-plus^®^ was obtained from Perrigo (Minneapolis, MN, United States). 2HF was prepared in dimethyl sulfoxide (DMSO) and diluted in Ora-plus as the solvent concentration did not exceed 0.2% (v/v) in the final solution. In the vehicle sample (absence of 2HF), a similar volume of DMSO (2% v/v) was added. The ophthalmic solution of tetracaine hydrochloride (1%) and phenylephrine hydrochloride (0,1%) was purchased from Allergan Aesthetics (Dublin, Ireland).

### 2.2 Animals and ethics statement

Female BALB/c mice (10–12 weeks; provided by the Instituto Ciências e Tecnologia em Biomodelos, ICTB/FIOCRUZ) were used in this study. All animals were bred and maintained at the Oswaldo Cruz Foundation according to the Guide for the Care and Use of Laboratory Animals of the Brazilian National Council of Animal Experimentation (COBEA). This study was performed in strict accordance with the recommendations of the Guide for the Care and Use of Laboratory Animals of the Brazilian National Council of Animal Experimentation (COBEA). The protocol was approved by the Committee on the Ethics of Animal Experiments of the Instituto Oswaldo Cruz (CEUA-IOC, License Number: L-11/2017 A2).

### 2.3 Method development

#### 2.3.1 High-performance liquid chromatography

Chromatographic separation was carried out on a prominence liquid chromatography system (Shimadzu^®^, Kyoto, Japan) with an HTS PAL automatic injector (CTC Ana-lytic, Zwingen, Switzerland). Chromatographic analysis was performed on a Zorbax Eclipse C_18_ analytical column (5 μm, 150 × 4.6 mm i.d.—Agilent Technologies^®^, CA, United States) in isocratic mode. The mobile phase consisted of acetonitrile and methanol (80:20, v/v) and an aqueous solution of formic acid 0,1% (65:35, v/v) at a flow rate of 0.8 mL/min. The injection volume was 20 µL. The temperature of the autosampler and column oven were 10 °C and 40 °C, respectively. The run time was 5.50 min (Supplementary Table S1).

#### 2.3.2 Mass spectrometry

Mass spectrometry analysis was conducted using an Applied API 4000 system (AB Sciex, United States), a linear triple quadrupole system with an electrospray ion source operating in negative ionization mode (ESI-). The ion spray voltage was −4500 V, and the source temperature was 500 °C. Nitrogen (N_2_) was used as a nebulizer and auxiliary gas. Collision-activated dissociation (CAD) gas and curtain gas at 6 and 15 psi, respectively (Supplementary Table S2). Mass spectrometry detection was performed using multiple reaction monitoring (MRM) of the transitions selected m/z 239.0 → 119.0 and 239.0 → 93.0 for 2′-hydroxyflavanone (2HF) and m/z 270.9 → 151.0 and 270.9 → 119.0 for naringenin, the internal standard (IS). The two transitions were used to identify 2HF and IS but only the most intense was used to quantify them. The collision energies for 2HF and IS were −23 and −26 V, respectively. All the individual parameters are presented in Supplementary Table S3. Data acquisition and processing were performed using Analyst TM software version 1.6.1 (AB Sciex^®^, CA, United States).

#### 2.3.3 Stock and working solution preparation

Standard stock solutions of 2HF and IS were prepared by dissolving the analytical standard in 100% methanol at a concentration of 1 mg/mL. The working solutions were prepared by successive dilutions of the stock solution in ultrapurified water and methanol (80:20 v/v). As the volume of mouse blood used in the method was very small and limited, we decided to use the standard addition method in which the blood concentrations in the calibration curve and quality controls obtained were 1, 3, 5, 25, 50, 100, 200 and 250 ng/mL for 2HF and 250 ng/mL for IS. All standard working solutions and quality control samples were stored at −70 °C.

#### 2.3.4 Sample preparation

Fifty microliters of IS solution (250 ng/mL naringenin) was added to 100 µL of the BALB/c mouse blood sample containing 2HF in a 2 mL polypropylene tube. The samples were mixed by vortexing for 10 s 2HF and IS were extracted from blood using a 1,500 µL TBME 100% solution (liquid/liquid extraction) under vortex agitation for 10 min, followed by centrifugation at 19,900 × g for 5 min at 10°C. One milliliter of the organic phase was transferred to another propylene tube and evaporated using N_2_ gas at 50°C. The residue was resuspended with 0.300 mL of a solution composed of 35% water containing 0.1% formic acid and 65% acetonitrile and methanol (80:20, v/v) and mixed under vigorous shaking for 20 s. Aliquots (20 μL) from the final extract were injected into the HPLC–MS/MS system.

### 2.4 Method validation

The validation process was performed following the tests below.

#### 2.4.1 Selectivity test

Samples of the biological matrix, which were free of internal standards and analytes and obtained from 6 (six) different sources, were analyzed. The responses of interfering peaks close to the retention time of the analyte, which was below 20% for processed samples from the LLOQ (lower limit of quantification), were compared. The interfering peak responses close to the IS retention time were less than 5% of the IS response.

#### 2.4.2 Linearity

The linearity range encompassing the lower limit of quantification (LOQ) and the upper limit of quantification (ULOQ) of the calibration curve was defined while developing the bioanalytical method. The linear regression model used was heteroscedastic, demonstrating an increase in the standard deviation as a function of the increase in the calculated blood concentration. For this test, the ratio of the areas of the analyte and the internal standard (referring to the first calibration curve of the bioanalytical validation) was used. The choice of the most appropriate weighting factor was performed according to the method with the smallest sum of the relative errors, which was determined from the nominal values of the calibration standards versus their values obtained by the curve equation (% RE). The weighted method chosen was 1/X^2^.

#### 2.4.3 Precision and accuracy

The precision and accuracy tests included sample dilution tests in a biological matrix for possible validation of the quantification of samples with concentration values above the upper limit of quantification in the calibration curve (ULOQ). The samples were diluted using 90% of the biological matrix and mouse blood, which was free of analyte and internal standard, and 10% of the biological matrix and mouse blood, which were added to the analyte for dilution. After mixing the specified volumes, the samples were stirred to homogenize the mixture and were subsequently aliquoted to obtain the working volume. Then, an internal standard was added, followed by the sample extraction process. Accuracy is expressed as relative standard error (RSE) and was calculated from Eq. 1 as follows:

$$RSE=\left[\left(Mean\;experimental\;concentration-nominal\;value\right)\times 100\right]÷nominal\;value$$ (1)

Precision is expressed as % CV and was calculated from Eq. 2 as follows:

$$CV=\left(Standard\;deviation\times 100\right)÷mean\;experimental\;concentration$$ (2)

#### 2.4.4 Matrix effect

For this study, the specific biological matrix used was mouse blood, and 6 (six) samples from different sources (four normal and two lipemic) were utilized for the analyses. For each sample, the IS-normalized matrix factor (MF) was obtained. The area responses obtained from the blank samples of biological matrices submitted to the extraction process were analyzed, and analytes and internal standards were subsequently added to verify the effect of the biological matrix as well as the effects of samples without the biological matrix (100% samples in solution). The IS-normalized matrix factor was calculated from Eq. 3 as follows:

$$MF=\frac{\left(Analyte\;area\;in\;matrix÷IS\;area\;in\;matrix\right)}{\left(Analyte\;area\;in\;solution÷IS\;area\;in\;solution\right.}$$ (3)

#### 2.4.5 Stability (freeze-thaw. Long-term, postpreparative and short-term)

Stability studies were initiated and completed before analyzing the studied samples. The stability in the biological matrix was evaluated through freezing and thawing cycles, short-term and long-term stability, and postpreparative. All tests were performed with eight replicates for each sample concentration level. To carry out the stability tests, an initial quantification test (TQI) was performed in accordance with local regulatory norms that aim to guarantee good practices through quality standards. In this test, samples of LQC (low-quality curve) and HQC (high-quality curve) were analyzed, in which the concentration was determined by utilizing a freshly prepared calibration curve. The number of replicates that were analyzed in each control was the same as that used in the stability tests. Freeze–thaw cycle temperatures are presented in Supplementary Table S4.

### 2.5 Pharmacokinetic study

#### 2.5.1 Protocol

BALB/c mice, with six mice per group, received a single dose of 10 mg/kg 2HF that was solubilized in DMSO (2%) and suspended in Ora-plus^®^ (Perrigo, Minneapolis, MN, United States). ORA-Plus is an aqueous-based vehicle consisting of suspending agents that have a high degree of colloidal activity, facilitating the process involved in the extemporaneous compounding of oral suspensions. Ora-Plus contains purified water, microcrystalline cellulose, sodium carboxymethylcellulose, xanthan gum, and carrageenan as suspending agents; calcium sulfate, sodium phosphate, and citric acid as buffering agents; simethicone as an antifoaming agent; and potassium sorbate and methylparaben as preservatives, as described in the datasheet. The doses were administered orally using a gavage needle.

#### 2.5.2 Sample collection

Mouse blood (300 µL) was collected *via* the orbital plexus, one mouse per time, with six replicates (6 mice per time interval). For anesthesia, an ophthalmic solution of tetracaine hydrochloride (1%) and phenylephrine hydrochloride (0,1%) was used, and one drop was administered in the chosen orbital 5 min before collection. The collection was performed with pasteurized Pasteur pipettes that were previously autoclaved, and blood was transferred to 1.5 mL microtubes that were also heparinized. The time intervals determined were 2.5, 5, 7.5, 10, 15, 30, 45, 60, 90, 120, and 240 min after administration of the compound. After collecting all samples, 2HF was extracted as described above.

#### 2.5.3 Pharmacokinetic parameters

The pharmacokinetic parameters were obtained by non-compartmental analysis (NCA) using the Phoenix WinNonlin^®^ 8.1 program (CERTARA). The maximum blood peak concentration (C_max_) and time to peak concentration (T_max_) were obtained directly from the blood concentration-time profiles. The area under the concentration-time curve from time zero to time t (AUC_0–t_) was obtained using the trapezoidal method. The area under the total blood concentration-time curve from time zero to infinity (AUC_0–∞_) was calculated using Eq. 4 as follows:

$$AUC_{0–\infty }=AUC_{0–t}+\left(C_{last}÷k_{el}\right)$$ (4)

where C_last_ is the concentration observed at the last time, and k_el_ is the apparent elimination rate constant obtained from the terminal slope of the blood concentration-time curve after logarithmic transformation of the blood concentrations and application of linear regression.

The blood elimination half-life (t_1/2_) was calculated using Eq. 5 as follows:

$$t\frac{1}{2}=\ln_{2}÷k_{el}$$ (5)

Apparent oral clearance (CL/F) and apparent volume of distribution (Vd/F) parameters were obtained from the blood concentrations and expressed normalized to bioavailability (F) because only oral data are available.

### 2.6 Statistical analysis

Statistical significance was tested using Phoenix WinNonlin^®^ 8.1 program (CERTARA) for the pharmacokinetics parameters.

## 3 Results

### 3.1 Method development

**FIGURE 2:**
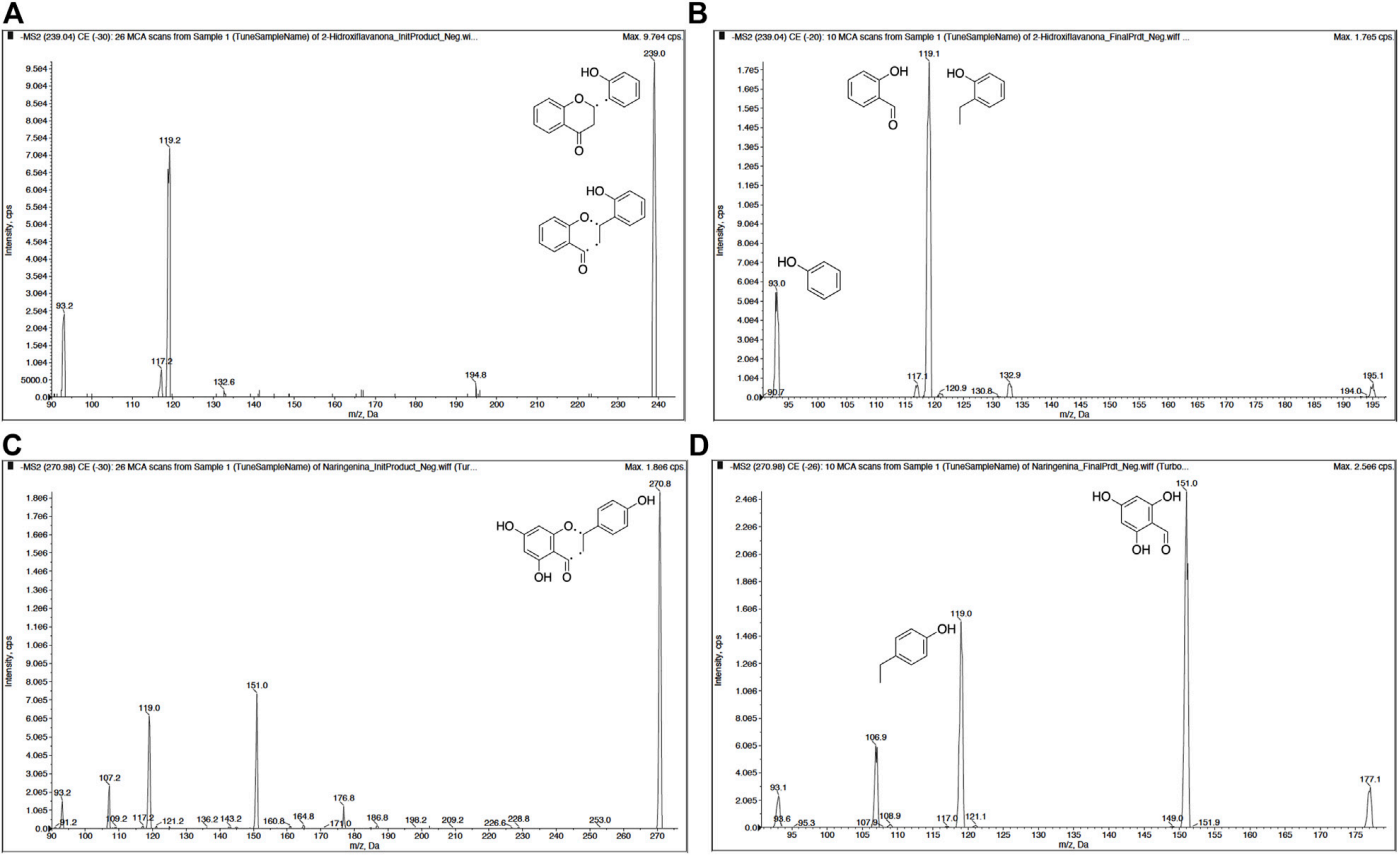
Product ion mass spectra of 2′-hydroxyflavanone (2HF) and Internal Standard (IS) in negative ionization mode. **(A)** The initial product of 2HF and **(B)** the final product of 2HF with transitions m/z 239.0 → 119.0 and m/z 239.0 → 93.0. **(C)** The initial product of IS and **(D)** the final product of IS with transitions m/z 270.9 → 151.0 and m/z 270.9 → 119.0. Dashed bonds in structures in **(A)** and **(C)** indicate the possible fragmentation point.

**FIGURE 3:**
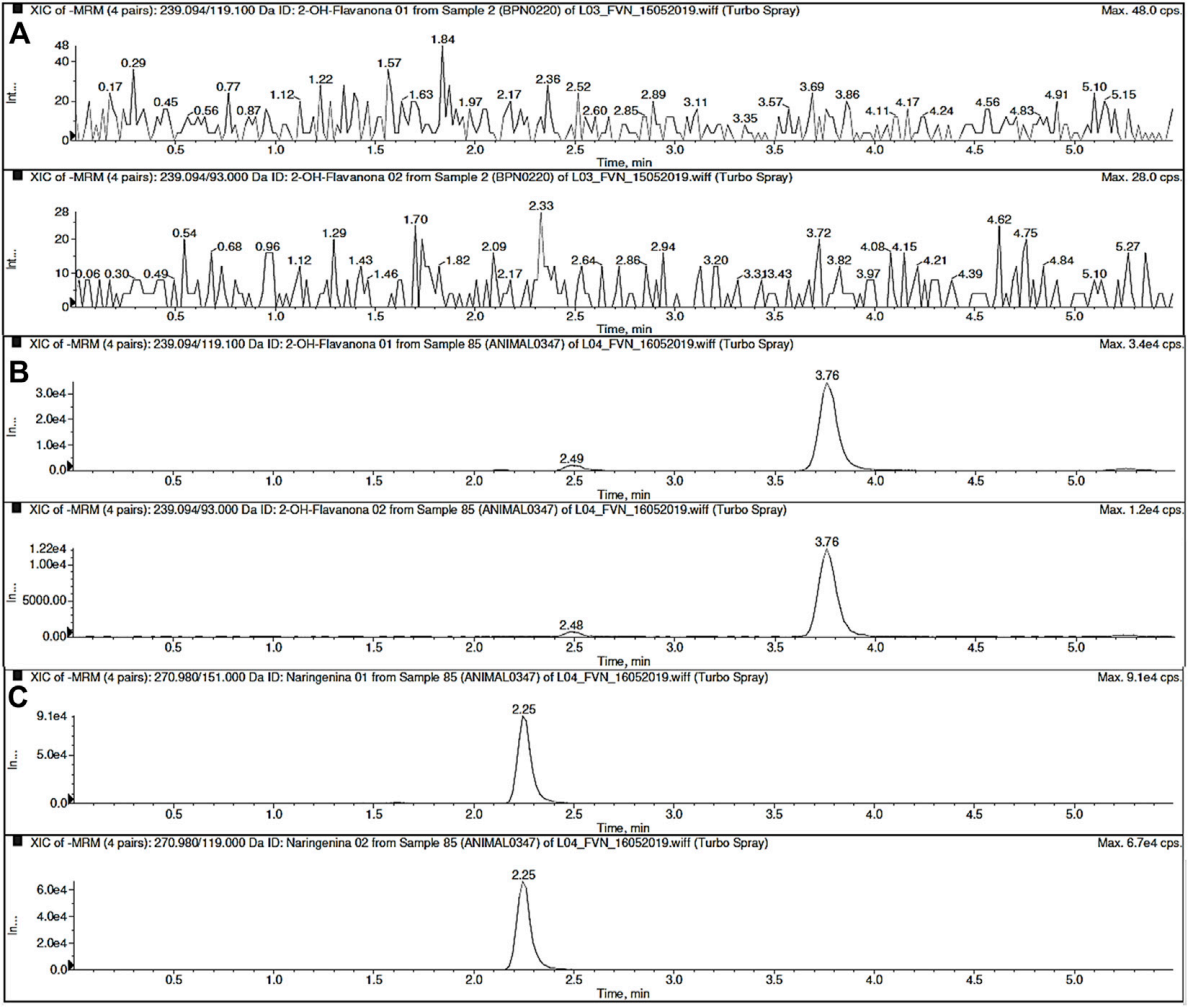
Representative MRM chromatograms of blank sample **(A)** and 2′-hydroxyflavanone (2HF) **(B)** and Internal Standard (IS; naringenin) **(C)** in blood sample.

To detect 2HF in BALB/c mouse blood, a sensitive and selective method was developed using HPLC–MS/MS. Narigenin, which is also a flavanone, was used as an IS. MS parameters were optimized using a direct infusion in a syringe pump in the flow of 20 μµL/min for 2HF or IS. The ion product spectrum was obtained in electrospray negative product ion mode using multiple reaction monitoring (MRM). The transitions selected for detection were m/z 239.0 → 119.0 and m/z 239.0 → 93.0 for 2HF and m/z 270.9 → 151.0 and m/z 270.9 → 119.0 for IS and the most abundant was used to quantify both 2HF and IS (Figures 2, 3).

**FIGURE 4:**
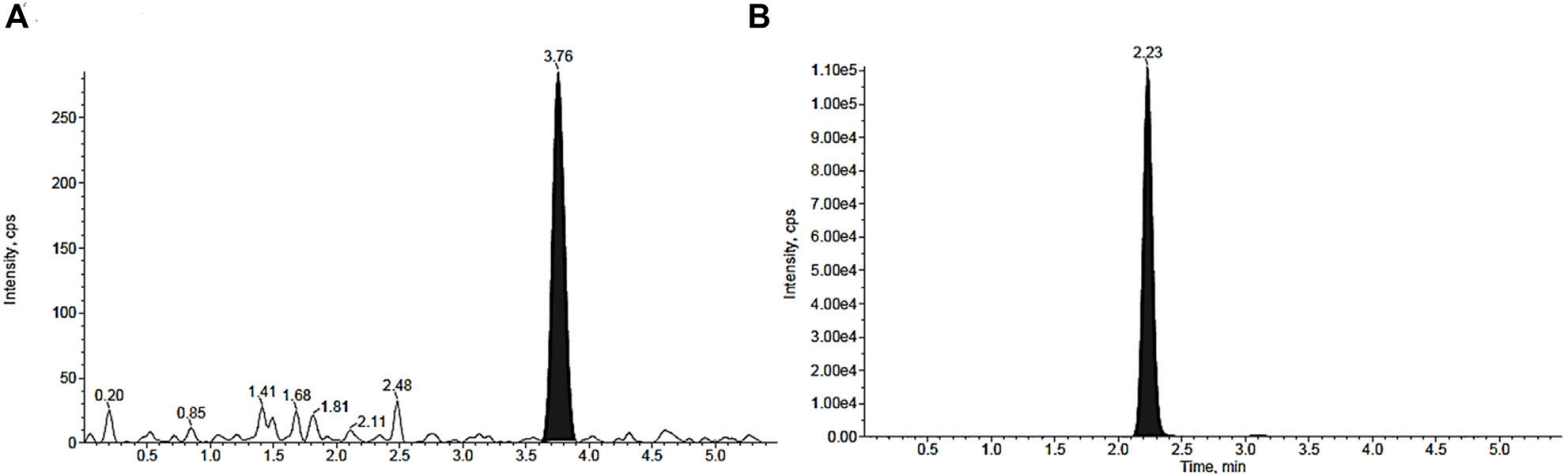
MRM chromatograms of blood samples spiked with 2′-hydroxyflavanone (2HF) **(A)** and Internal Standard (IS; naringenin) **(B)**, demonstrating retention times of 3.76 min and 2.23 min, respectively.

After the mass spectrometry parameters were set, the best chromatography analysis conditions were achieved using a mixture of 35% aqueous solution with formic acid (0,1%) and 65% acetonitrile and methanol (80:20, v/v) with a C_18_ analytical column. 2HF and IS were extracted from BALB/c mouse blood using liquid–liquid extraction (LLE), and the established concentration range was from 1 ng/mL (lower limit of quantification–LLOQ) to 250 ng/mL (upper limit of quantification–ULOQ). Naringenin was selected as the internal standard (IS) because its chemical properties are similar to those of 2HF. Both the analyte and IS showed different retention times, which were 3.76 and 2.23 min, respectively (Figure 4).

### 3.2 Method validation

#### 3.2.1 Selectivity test

The analysis of six different BALB/c mouse blank blood samples (two lipemic and four normal) was performed to determine the specificity. No interfering peaks were observed at the retention times for 2HF and IS, as shown in Supplementary Table S5.

#### 3.2.2 Linearity and limit of quantification

**FIGURE 5:**
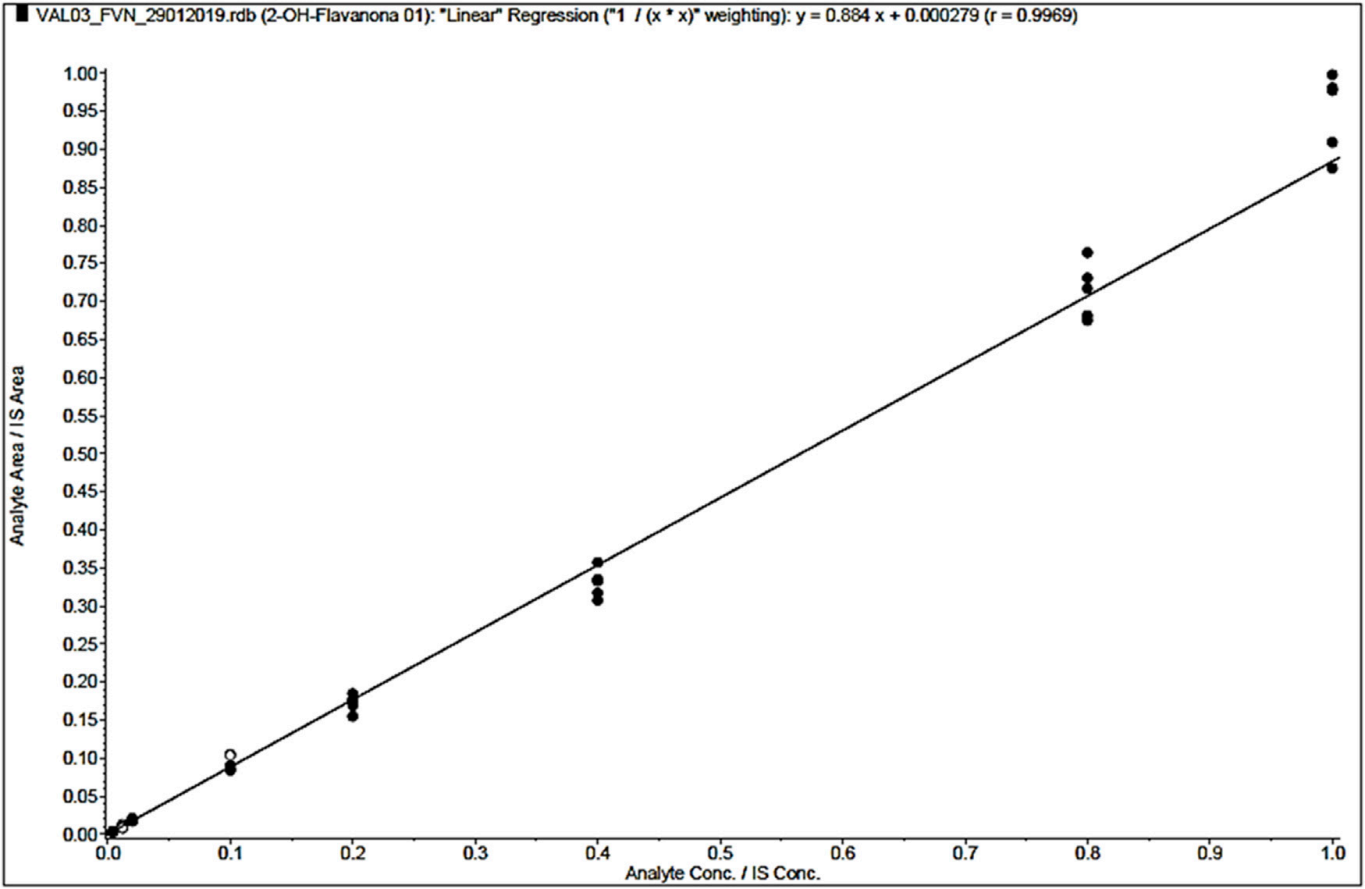
2′-hydroxyflavanone (2HF) linearity curve: Representation of the values of the calibration curve with the coefficients with the chosen weight (model 1/X^2^). The calibration range was established at 1, 3, 5, 25, 50, 100, 200, and 250 ng/mL.

The lower limit of quantification (LLOQ) was determined to be 1 ng/mL. The calibration range was established at 1–250 ng/mL. The calibration curves were fitted using linear regression, with a weight factor of 1/X^2^ (X = concentration), a method that covers all clinical concentrations of 2HF. A correlation coefficient of 0.9969 was recorded during the validation study (Figure 5).

#### 3.2.3 Precision and accuracy test

Precision and accuracy tests of the bioanalytical method were performed on three consecutive days. Intra- and interday precision and accuracy were calculated using eight replicates for each concentration level (LLOQ: 1, LQC: 3, MQC: 100, HQC: 200 and DQC: 2000 (diluted to 200) ng/mL). The results obtained in each test were approved according to the criteria established (Table 1).

**TABLE 1:** Precision and accuracy for intraday and interday.

Normal concentration (ng/mL)	LLOQ	LQC	MQC	HQC	DQC (2000.00 diluted to 200.00)
	1.00	3.00	100.00	200.00	200.00
Day 01					
Mean	0.85	2.62	87.07	201.75	200.59
S.D. (+/−)	0.10959	0.13141	12.56256	8.78316	11.11895
C.V. (%)	12.82	5.01	14.43	4.35	5.54
Accuracy (%)	85.50	87.39	98.07	100.87	100.29
Day 02					
Mean	0.98	2.79	101.15	212.18	210.12
S.D. (+/−)	0.06955	0.40814	3.68255	4.59690	4.20608
C.V. (%)	7.07	14.65	3.64	2.17	2.00
Accuracy (%)	98.35	92.85	101.15	106.09	105.06
Day 03					
Mean	1.02	3.02	98.47	201.49	199.34
S.D. (+/−)	0.06298	0.20430	1.86319	6.01864	3.21374
C.V. (%)	6.18	6.76	1.89	2.99	1.61
Accuracy (%)	101.96	100.77	98.47	100.75	99.67
Interday (n = 3 days)					
Mean	0.95	2.81	95.56	205.14	203.35
S.D. (+/−)	0.08654	0.20176	7.47908	6.10079	5.89743
C.V. (%)	9.08	7.18	7.83	2.97	2.90
Accuracy (%)	95.27	93.67	95.56	102.57	101.67

LLOQ, lower limit of quantification; MQC, medium-quality curve; LQC, low-quality curve; HQC, high-quality curve; DQC, dilution-quality curve; S.D., Standard deviation; C.V., variance coefficient.

#### 3.2.4 Matrix effect

The matrix effect assay was performed using two concentration levels (LQC: 3.00 ng/mL and HQC: 200.00 ng/mL). The ratio between the relative responses of the analyte and the internal standard for each sample analyzed was used to calculate the normalized matrix factor as well as variance coefficient (C.V. %). The obtained results presented C.Vs. that fit the criteria, indicating that there is no significant interference of the matrix components in the extraction of 2HF (Supplementary Tables S6, S7; Table 2).

**TABLE 2:** Matrix effect test results.

Normal concentration (ng/mL)	NMF	Mean	Total C.V. (%)
3 (LQC)	0.96	0.96	2.91
	0.95		
	0.94		
	0.97		
	1.01		
	0.93		
200 (HQC)	0.91	0.96	2.64
	0.94		
	0.97		
	0.97		
	0.98		
	0.98		

LQC, low-quality curve; HQC, high-quality curve.

NMF, normalized matrix factor; C.V., variance coefficient.

#### 3.2.5 Stability

The stability of 2HF in BALB/c mouse blood was evaluated using short-term tests at 22 °C, postprocessing stability at +10 °C, stability in freeze–thaw cycles (n = 06 cycles) and long-term stability with the samples stored at −70 °C. In these assays, the quality controls of low and high concentrations, LQC: 3.00 ng/mL and HQC: 200.00 ng/mL, were used. In all stability tests performed, no significant degradation of the 2HF analyte was observed (Table 3).

**TABLE 3:** Stability tests of the 2′-hydroxyflavanone (2HF) in mouse blood under different storage and exposure conditions (*n* = 8).

Normal concentration (ng/mL)	LQC	HQC
	3.00	200.00
Short term stability (+22.0 °C)		
Mean	3.14	215.06
S.D. (+/−)	0.18249	9.86729
C.V. (%)	5.82	4.59
Deviation (%)	4.59	7.53
Postpreparative stability (+10.0°C)		
Mean	3.30	222.16
S.D. (+/−)	0.16216	9.66433
C.V. (%)	4.91	4.35
Deviation (%)	10.11	11.08
Freeze–thaw stability (−70.0°C)		
Mean	3.05	209.37
S.D. (+/−)	0.16799	13.07105
C.V. (%)	5.51	6.24
Deviation (%)	1.61	4.68
Long-term stability (−70.0°C)		
Mean	2.96	210.83
S.D. (+/−)	0.09308	7.72332
C.V. (%)	3.14	3.66
Deviation (%)	−1.33	5.42

LQC, low-quality curve; HQC, high-quality curve; DQC, dilution-quality curve; S.D., Standard deviation; C.V., variance coefficient.

### 3.3 Pharmacokinetic study

**FIGURE 6:**
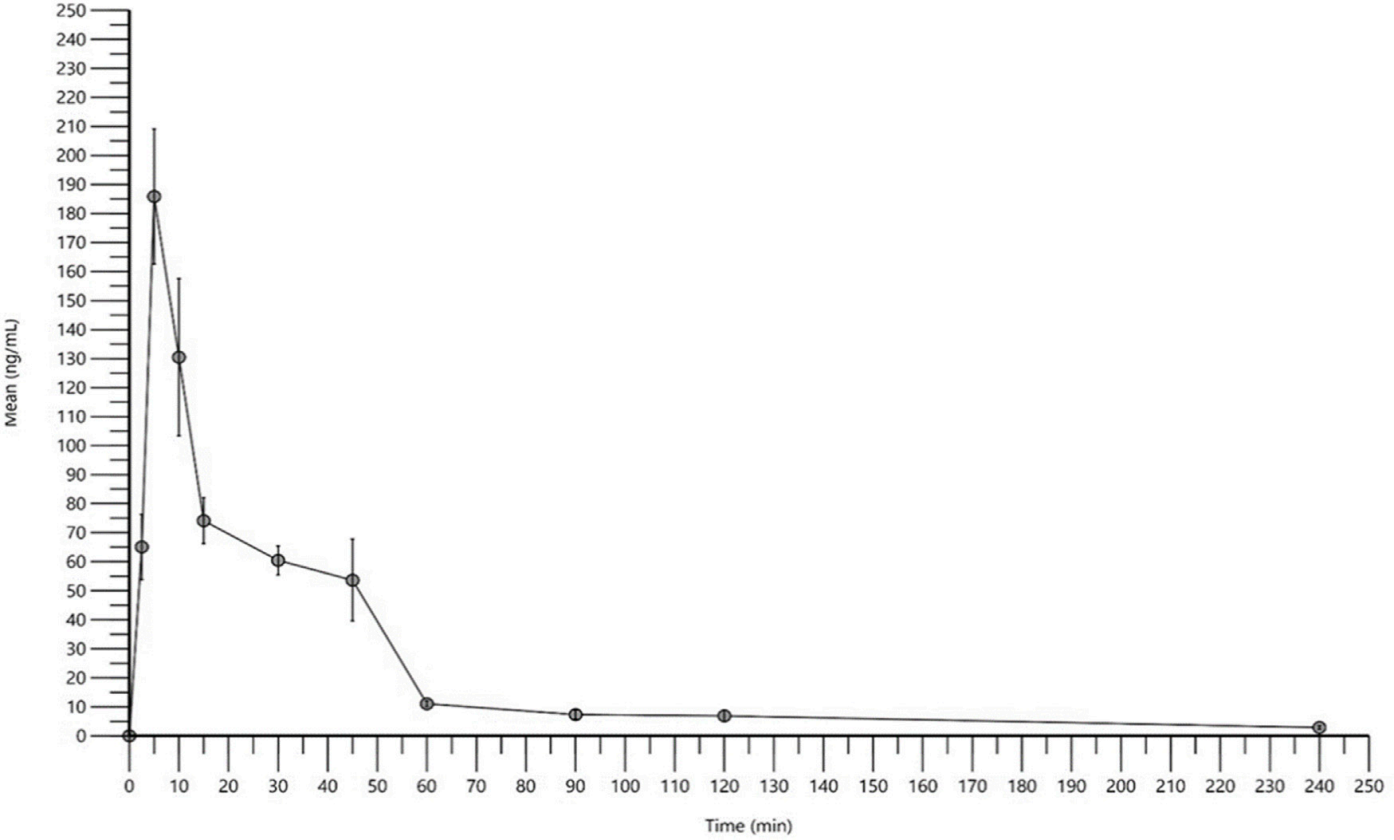
2′-hydroxyflavanone (2HF) pharmacokinetic profile. Blood concentration versus time. BALB/c mice were orally treated with 10 mg/kg of 2HF. Mouse blood was collected *via* the orbital plexus for detection and quantification by the validated 2HF method through HPLC–MS/MS. The values plotted on the graph are the mean and standard error (*n* = 6).

Once developed and validated, the 2HF detection and quantification method was used to determine the 2HF BALB/c mouse blood concentration versus time curve. Six female BALB/c mice per group (each time interval) received a single dose of 2HF at 10 mg/kg. The time intervals determined were 2.5, 5, 10, 15, 30, 45, 60, 90, 120 and 240 min. Mean blood concentration values were calculated for each time interval. A graph was constructed with the mean values of blood concentration as a function of time (Figure 6).

Pharmacokinetic parameters were determined by a non-compartmental model. 2HF exhibited a C_max_ of 185.86 ng/mL with a T_max_ of 5 min and a half-life (T_1/2_) of 97.52 min (Table 4).

**TABLE 4:** Pharmacokinetic parameters of 2′-hydroxyflavanone (2HF).

Parameters (units)	Values
Kel (1/min)	0.00711
T_1/2_ (min)	97.52
T_máx_ (min)	5
C_máx_ (ng/mL)	185.86
AUC_0-t_ (min x ng/mL)	5,120.98
AUC_0-inf_ (min x ng/mL)	5,524.54
AUC % extrap (%)	7.30
Vd/F (mL)	2,546.76
Cl/F (mL/min)	18.10

Kel, elimination rate constant; T_1/2_, elimination half-time; T_max_, time to reach C_max_; C_max_, maximum concentration; AUC, area under the curve; Vd/F, volume of distribution; Cl/F, total blood clearance.

## 4 Discussion

Natural products have been a powerful source of therapeutic agents for traditional medicine since ancient times. Over time, pharmacology studies have begun to center the discovery and development of drugs on chemical structures and metabolite isolations.

Natural products are known for their antiviral, anti-inflammatory, antineoplastic, trypanosomicidal and leishmanicidal activities (Ioset, 2008; Gervazoni et al., 2020). Despite the natural abundance, therapeutic properties and low cost of natural products, it seems that promising studies on natural products are not being continued, especially trypanosomicidal studies (Schmidt et al., 2012a; 2012b; Gervazoni et al., 2020).

Research on 2′-hydroxyflavanone (2HF) has been performed with several types of cancer and has shown promising and selective results (Hsiao et al., 2007; Nagaprashantha et al., 2011; 2018; 2019; Shin et al., 2012; Singhal et al., 2015; Bose et al., 2019; Yue et al., 2020). Recently, 2HF was identified as a good leishmanicidal candidate drug (Gervazoni et al., 2018), which indicates the need for further investigations and further drug development steps, such as preclinical studies with animal studies and establishing drug pharmacokinetics (Singh et al., 2012; Huang et al., 2017).

According to the literature, a good drug candidate must exhibit good pharmacokinetic properties (Addona et al., 2009; An et al., 2014). To obtain the pharmacokinetic parameters, it is necessary to develop a detection method for the desired compound. Thus, a bioanalytical method was developed for 2HF in HPLC–MS/MS. HPLC–MS/MS has been described as a selective, sensitive, and efficient method for drug development (Addona et al., 2009; An et al., 2014; Becher et al., 2017).

In the development of the method, we observed that using acid in the mobile phase presented better ionization results in the negative mode. Although acid is generally used in positive mode ionization, adding weak acids in the mobile phase can have a more favorable signal-to-noise ratio effect than using a base in the mobile phase in negative mode ionization (Wu et al., 2004).

This method allowed 2HF to be sensitively detected in a range comprising the lower and upper limits of quantification (LLOQ and ULOQ) from 1 to 250 ng/mL. In this study, naringenin was used as an internal standard (IS). 2HF and Naringenin belong to the same flavonoid class, with similar chemical structures, and demonstrated similar HPLC–MS/MS parameters and retention times, 3.76 and 2.23 min for naringenin and 2HF, respectively (Martins et al., 2000; 2013).

Selectivity was the first test to validate the method. 2HF did not show any interference peaks compared to the low interference demonstrated by the pertuzumab surrogate peptide (Schokker et al., 2020), but both comprised the acceptance criteria. Method linearity was achieved by preparing matrix-matched calibration curves. The calibration curves were fitted using linear regression with a weighting factor of 1/X^2^, and a coefficient value of r = 0.9969 was obtained. Using the same linear method, Li et al. (2020) evaluated nine compounds with HPLC–MS/MS and obtained a coefficient value over 0.99, which was very similar to that of 2HF (Li et al., 2020).

The intraday and interday precision and accuracy were assessed at the four quality curve levels (QC). The accuracy for all QCs in both assays was over 95%, and the precision fit the acceptance criteria guidance. The matrix effect was also evaluated and indicated that the bioanalytical method did not suffer from interference by matrix components in the 2HF analysis in mouse blood. The evaluation was performed using two different matrices. Tikhomirov et al. (2021) demonstrated that lipemia in plasma samples affected the fentanyl quantification by HPL-MS/MS, indicating the importance of the matrix effect analysis and with different matrices.

Short-term, postprocessing, long-term and freeze–thaw assays were performed to assess the 2HF matrix stability. Stability tests performed under different exposure conditions of the 2HF analyte contained in mouse blood showed that there was no significant degradation either at room temperature or at the storage temperature of the samples during the entire study period.

Once optimized and validated, the method was applied to detect 2HF in BALB/c mouse blood at different times to demonstrate its pharmacokinetic properties. After administering a single dose of 10 mg/kg 2HF orally to BALB/c mice, blood was collected to quantify the 2HF concentration at different collection times. We observed that the chosen collection points allowed a good characterization of the pharmacokinetic profile for the 2HF, with well characterized absorption, distribution, and elimination phases. This was important for an adequate determination of the pharmacokinetic parameters, since the parameters were directly extracted from the concentration-time curve using a non-compartmental analysis. However, further points might be necessary in the absorption phase to elucidate other pharmacokinetic parameters, but unfortunately, for operational reasons, it was not possible.

To calculate the pharmacokinetic parameters, a non-compartmentalized analysis was performed. We observed that 2HF reached its maximum concentration peak in 5 min (T_max_), with a C_max_ of 185.86 ng/mL. The half-life (t_1/2_) determined for 2HF was 97.52 min. Half-life is a very important parameter that is obtained when studying the pharmacokinetics of a compound. It is an elimination pharmacokinetic parameter that can assist in estimating the duration of drug action as well as in establishing dosing regimens. Regarding the short half-life observed, an oral administration of 2HF could not reach the blood concentration levels necessary for a steady state in an 8-, 12- or 24-hour dose model (longer times of administering medications). However, as demonstrated by the *in vivo* activity of 2HF in an experimental model of cutaneous leishmaniasis, a significant effect was observed with daily doses of 50 mg/kg for 35 days, indicating that even with a short half-life, 2HF promoted the expected effect (Gervazoni et al., 2018).

It can also be observed in cancer studies. 2HF treatment inhibited different types of tumor growth with single oral doses of 100 mg/kg/day (Yue et al., 2020) or 25, 50 and 100 mg/kg every other day (Nagaprashantha et al., 2011; Singhal et al., 2018a; Singhal et al., 2018b). Furthermore, apigenin and quercetin, both flavonoids with a short T_1/2_ (less than 192 min), have promising *in vivo* effects in a single oral dose/day cutaneous leishmaniasis model (Gervazoni et al., 2020).

Phloretin and cardamonin, both chalcones, had their oral pharmacokinetics in rat plasma determined by HPLC–MS/MS, with single doses of 100 mg/kg and 5 mg/kg, respectively (Jaiswal et al., 2017; Zhao et al., 2020). Phloretin analysis described a T_max_ (h) of 1.25 and T_1/2_ (h) of 2.83. Cardamonin demonstrated a T_max_ (h) of 0.50, a fast absorption that can correlate with the 2HF T_max_. The pharmacokinetic parameters of four flavonoids abundant in Hoveniae Semen, dihydromyricetin, dihydroquercetin, myricetin and quercetin, which were orally administered to rats, were evaluated. The T_max_ values obtained were 0.57 h, 0.28 h, 0.22 h, and 0.25 h for dihydromyricetin, dihydroquercetin, myricetin and quercetin, respectively, and T_1/2_ values were 3.04 h, 2.32 h, 2.46 h and 3.00 h for dihydromyricetin, dihydroquercetin, myricetin and quercetin, respectively (Yang et al., 2019a). Pharmacokinetic parameters for apigenin were determined, demonstrating a T_1/2_ value of 2.11 h, T_max_ value of 0.50 h and C_max_ value of 42 ng/mL (Teng et al., 2012). It has been described that a 10 mg/kg oral dose of flavokavain B when administered in rats demonstrated a C_max_ of 265.2 ng/mL in 1 h and a T_1/2_ of 2.76 h (Yang et al., 2019b). These results correlate with 2HF parameters since all the flavonoids cited have, compared to other substances, short values of T_max_ and T_1/2_.

Additionally, it is important to point out that administration *via* gavage should also be considered to explain why 2HF demonstrated a short T_max_. Gavage administration makes the compound available directly in the stomach, skipping the first stages of oral intake (Foster et al., 2015). Further *in vivo* studies can lead to a correlation of IC_50_ with the pharmacokinetic features of the flavonoid in leishmaniasis or cancer.

## 5 Conclusion

In this study, a sensitive and selective method for 2HF quantification was developed, achieving the detection of 2HF in mouse blood at different times after oral administration, leading to the determination of the pharmacokinetic parameters. The majority of leishmanicidal natural compounds have not undergone preclinical studies, and pharmacokinetic studies of flavonoids are poor; therefore, this study represents an important advancement for flavonoids. Since the literature indicates that 2HF is a promising candidate for different types of cancer treatments, our results can be used in a multidisciplinary way to help other research. However, formulation studies, such as nanoencapsulation of 2HF or the application of a drug-controlled delivery system, should be conducted.

## Data Availability

The original contributions presented in the study are included in the article/Supplementary Material, further inquiries can be directed to the corresponding author.
