# Supplementary material for: Analysis of 2′-hydroxyflavanone (2HF) in mouse whole blood by HPLC–MS/MS for the determination of pharmacokinetic parameters
Source: Front Chem. 2023 Mar 9;11:1016193. doi: 10.3389/fchem.2023.1016193 (PMC10033538; doi:10.3389/fchem.2023.1016193)
Supplement: Supplementary file 1 [file Table1.PDF]

Supplementary table 1. Chromatographic conditions

Parameters	Description
Analytical column	Zorbax Eclipse C 18 5 µm x 150 mm x 4.6 mm id
Mobile phase	Pump A: 35% water containing 0.1% formic acid<br>Pump B: 65% acetonitrile/ methanol (80:20, v/v)
Flow rate	0.800 mL/min
System pressure	96 bar
Autosampler temperature	10 °C
Injection volume	20 µL
Run time	5.50 min
Room temperature	22 ± 2 °C
Retention time	2HF: 3.74 min/ IS: 2.23 min

2HF 2'-hydroxyflavanone; IS - Internal Standard

Supplementary table 2. Mass Spectrometry conditions

Parameters (units)	Description
ESI ionization mode	Negative
Ionspray voltage (V)	- 4500
Source temperature (°C)	500
CAD gas (units)	6
Curtain gas (psi)	15
GS1 (psi)	45
GS2 (psi)	45

ESI – electrospray source ionization;

CAD – collision gas;

GS1 – nebulizing gas;

GS2 – heater gas

Supplementary table 3. Individual ions parameters

Parameters (units)	2HF	IS
Fragment transition (m/z)	239.0 → 119.0	270.9 → 151.0
Time (msec)	250	250
DP (V)	- 70	- 75
CE (V)	- 23	- 26
EP (V)	-10	-10
CXP (V)	- 1	- 9

DP - declustering potential; CE - collision energy; EP - entrance potential; CXP - collision cell exit potential; 2HF - 2'-hydroxyflavanone; IS - Internal Standard.

Supplementary table 4. Freeze–thaw cycles temperatures

Cycles	Thaw temperature (°C)	Freeze temperature (°C)
Freeze	NA	-74.4
1st cycle	22.9	-73.7
2nd cycle	23.6	-74.6
3rd cycle	23.1	-74.2
4th cycle	23.1	-74.0
5th cycle	23.6	-75.5
6th cycle/analysis	22.4	NA

Supplementary table 5: 2'-hydroxyflavanone selectivity test

Sample description	2HF area value	2HF % of interference	IS area value	IS % of interference
**Blank in lipemic blood**	0	0	0	0
**LLOQ in lipemic blood**	2942.12		593458.87	
**Blank in normal blood 1**	0	0	0	0
**LLOQ in normal blood 1**	3169.71		614016.00	
**Blank in normal blood 2**	0	0	0	0
**LLOQ in normal blood 2**	3420.71		675769.91	
**Blank in normal blood 3**	0	0	0	0
**LLOQ in normal blood 3**	3926.56		566784.50	
**Blank in normal blood 4**	0	0	0	0
**LLOQ in normal blood 4**	4005.95		747538.16	
**Blank in normal blood 5**	0	0	0	0
**LLOQ in normal blood 5**	3938.97		761398.91	

LLOQ – Lower limit of quantification; 2HF - 2'-hydroxyflavanone; IS - Internal Standard

Supplementary Table 6. 2'-hydroxyflavanone and Internal Standard area values in matrix

Normal concentration (ng/mL)	Matrix type	2HF area values	IS area values	2HF area/IS area ratio	Mean
**3 (LQC)**	Lipemic blood 1	18189.40	1118085.79	0.01627	0.01618
	Lipemic blood 2	18305.91	1148566.24	0.01594	
	Normal blood 1	19103.09	1206115.03	0.01584	
	Normal blood 2	19434.52	1186209.50	0.01638	
	Normal blood 3	19138.78	1127579.97	0.01697	
	Normal blood 4	18868.64	1203852.84	0.01567	
**200 (HQC)**	Lipemic blood 1	1217165.39	1174020.98	1.03675	1.08645
	Lipemic blood 2	1172966.69	1094521.67	1.07167	
	Normal blood 1	1162212.53	1052889.51	1.10383	
	Normal blood 2	1204290.28	1094114.45	1.10070	
	Normal blood 3	1043244.41	934243.89	1.11667	
	Normal blood 4	1019637.66	936193.94	1.08913	

LQC – low-quality curve; HQC – high-quality curve; 2HF - 2'-hydroxyflavanone; IS - Internal Standard;

Supplementary Table 7. 2'-hydroxyflavanone and Internal Standard. area values in solution

Normal concentration (ng/mL)	2HF area values	IS area values	2HF area/IS area ratio	Mean
**3 (LQC)**	18812.55	1125516.12	0.01671	0.01687
	19335.48	1069928.66	0.01807	
	19529.59	1184922.81	0.01648	
	19190.46	1163327.67	0.01650	
	18761.43	1102750.01	0.01701	
	19429.08	1183701.15	0.016141	
**200 (HQC)**	1224996.17	1116029.71	1.09764	1.13724
	1165602.49	1027349.87	1.13457	
	1110050.43	949415.46	1.16919	
	1112123.03	984674.60	1.12943	
	1012097.29	909941.55	1.11227	
	1053292.71	892387.11	1.18031	

LQC – low-quality curve; HQC – high-quality curve; 2HF - 2'-hydroxyflavanone; IS - Internal Standard;
